# Tuina for children with myopia: A protocol for a systematic review

**DOI:** 10.1097/MD.0000000000018342

**Published:** 2019-12-16

**Authors:** Jiao Rong, Huichao Feng, Jing Li, Mengmeng Wang, Tianjiao Lu, Xinghe Zhang, Qian Zhuang, Fujie Jing

**Affiliations:** a School of Acupuncture-Tuina, Shandong University of Traditional Chinese Medicine, Jinan; b Department of acupuncture and moxibustion, Weifang Hospital of Traditional Chinese Medicine, Weifang, Shandong, China.

**Keywords:** children, myopia, protocol, systematic, Tuina

## Abstract

**Background:**

The study aims to evaluate the effectiveness and safety of Tuina for children with myopia.

**Methods:**

The following electronic databases will be searched from establishment to July 2019: Cochrane Library, MEDLINE, EMBASE, Web of Science, Springer, World Health Organization International Clinical Trials Registry Platform (ICTRP), China National Knowledge Infrastructure (CNKI), Wan-fang database, Chinese Scientific Journal Database (VIP), Chinese Biomedical Literature Databases (CBM), and other databases. All published randomized controlled trials (RCTs) about this topic will be included. Two independent researchers will operate article retrieval, duplication removing, screening, quality evaluation, and data analyses by Review Manager (V.5.3.5). Meta-analyses, subgroup analysis, and/or descriptive analysis will be performed based on the included data conditions.

**Results:**

High-quality synthesis and/or descriptive analysis of current evidence will be provided from improvement of distant vision, improvement of myopic diopter, and side effects.

**Conclusion:**

This study will provide the evidence of whether Tuina is an effective and safe intervention for children with myopia.

**PROSPERO registration number:**

CRD42019142283.

## 1 Introduction

### 1.1 Description of the condition

Myopia is a group of disease presentation as spherical error of refraction caused by elongation of the eyeball or over accommodation. In myopic eye, the focus of a distant object is in front of the retina.^[1]^ The epidemic of myopia is a global public-health crisis in modern society. With increasing urbanization and lifestyle changes worldwide, there will be half of the world population, which is 4758 million people with myopia by the year 2050; and 938 million people with high myopia.^[2]^ Currently, 30% to 50% of adults in the United States and Europe are myopic, and the condition is much more prevalent in East Asians, affecting as many as 90% of high school students.^[3–5]^ Myopia has shown a significant increase in prevalence over the last 2 decades and has become one of the leading ocular disorders causing visual impairment worldwide.^[2,4,5]^

### 1.2 Description and function of intervention

Tuina (Chinese massage) is a physical therapy method that is based on Traditional Chinese Medicine (TCM) Zang-fu organs, meridian theory as the theoretical basis, combined with anatomical and pathological diagnosis in order to achieve dredging meridian and curative effect of harmonic Yin and Yang. Based on different meridian-acupoint theory and operation method, pediatric Tuina for children was separated from Tuina for adults in the Ming Dynasty.^[6,7]^ Clinical articles have been showed that Tuina can improve the patient's eye blood circulation, loosen eye muscle tension, relieve eye fatigue, and have a good impact on improving patient vision and improving myopia diopter.^[8,9]^ In many textbooks and clinical trials, teachers and doctors explained how to treat myopia by Tuina, such as rubbing Chengqi (ST1), rubbing Sibai (ST2), rubbing Jingming (BL1),pressing Guangming (GB37), pressing Xinshu (BL15), pushing Ganshu (BL18).^[10–14]^

### 1.3 Why the review is important

Childhood nearsightedness (myopia) is increasing in both incidence and severity, there is a concomitant increase in the risk of severe and irreversible loss of vision over time, including retinal detachment, subretinal neovascularization, early and dense cataracts, and glaucoma.^[15,16]^ In Chinese clinical trials, many treatments for children with myopia have drawn the method of Tuina treatment,^[17,18]^ However, the evidence was still limited based on nonstandard measurement, nonuniformed outcomes, subjectivity judgment, and other factors. On the other hand, no related review or protocol has been published. In order to evaluate the efficacy and safety of Tuina for myopia in children, it is necessary to conduct evidence-based review. So, this review is urgently needed to accomplish.

## 2 Methods

This systematic review protocol has been registered in the PROSPERO network (No. CRD42019142283). All steps of this systematic review will be performed according to the Cochrane Handbook (5.2.0).

### 2.1 Selection criteria

#### 2.1.1 Types of studies

Randomized controlled trial (RCT) and blinded research will be included. Published clinical trials that reported the efficacy and safety on Tuina for myopia will be included. RCTs that involve at least 1 Tuina related treatment to myopia, and 1 control treatment (or blank treatment) will be included. As there is a risk of interference with the outcome, nonrandomized controlled trials will be excluded. Studies of animal experiment, review, case report, and meta-analysis will be excluded.

#### 2.1.2 Types of patients

Patients who were diagnosed as myopia will be included, without limits on gender, age, race, nationality, and medical units; all children <18 years of age will be included in the study.

#### 2.1.3 Types of interventions and comparisons

Interventions can be any type of Tuina: acupressure, pushing, rubbing, transiting, stroking, and chiropractics based on meridian-acupoint theory of children. Multiple control interventions will be included: no treatment, placebo and other interventions (eg, acupuncture, moxibustion, drugs, physical interventions, gentle touch, and other massage therapies). Comparisons contain pediatric Tuina and its relation will be excluded. Interventions of pediatric Tuina combined with other therapies will also be included, only if these combinations are compared to the other therapies semplice.

#### 2.1.4 Types of outcomes

Primary outcomes will include improvement of distant vision; improvement of Myopia diopter, and side effects of Tuina. Secondary outcomes will include improvement of visual fatigue; symptom improvement; and degree of satisfaction with the treatment.

### 2.2 Search methods for identification of studies

#### 2.2.1 Electronic searches

The following electronic databases will be searched from establishment to July 2019: Cochrane Library, MEDLINE, EMBASE, Web of Science, Springer, World Health Organization International Clinical Trials Registry Platform (ICTRP), China National Knowledge Infrastructure (CNKI), Wan-fang database, Chinese Scientific Journal Database (VIP), Chinese Biomedical Literature Databases (CBM), and other databases. All published RCTs about this topic will be included. Exemplary search strategy of MEDLINE is listed in Table 1, terms are conform to medical subject heading (MeSH). According to the difference of databases, keywords may combined with free words and comprehensive search will be performed.

**Table 1:**
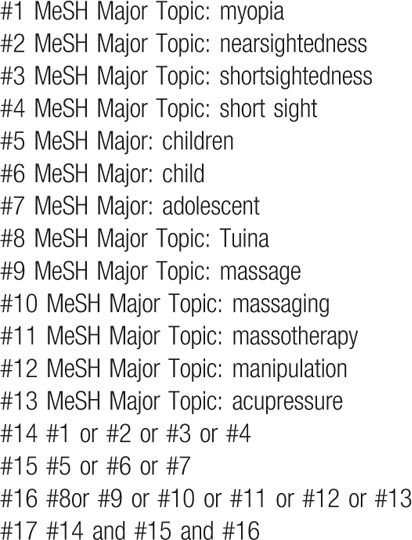
MEDLINE search strategy.

### 2.3 Data collection and analysis

#### 2.3.1 Selection of studies

**Figure 1:**
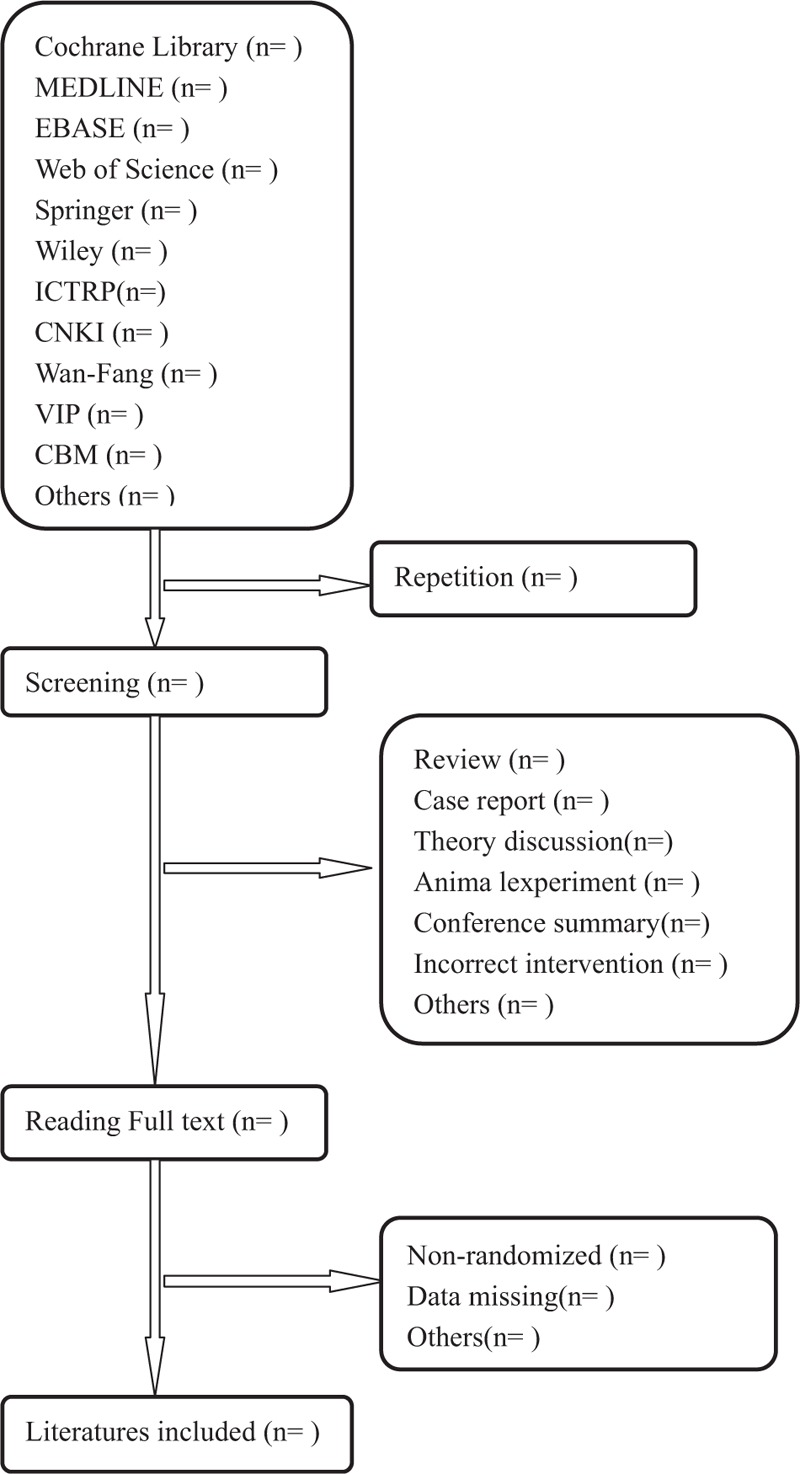
Flow diagram of studies identified.

Two authors (JR and HCF) will select clinical trials depending on inclusion criteria. After the title and abstract are screened, literatures that are not related and do not meet the criteria will be excluded. Screening operation will flow the diagram of Figure 1. If the full literatures are unable to obtained or related data is incomplete, we will contact the corresponding author. Third-party experts will be consulted to determine the selection divergence.

#### 2.3.2 Assessment and quality of included studies

Two authors (JL and XHZ) will evaluate quality of included articles and assess the risk of bias based on Cochrane Handbook 5.2.0. Quality assessment of included studies contains randomized method, allocation concealment, blinding of participants and personnel, blinding of outcome assessment, completeness of outcome data, and selective reporting. Divergence of evaluation will also consult third-party experts.

#### 2.3.3 Data extraction

The authors (JR and TJL) plan to extract the data from the articles selected for inclusion, and to resolve differences in opinion through discussion with experts. Data will be recorded onto an electronic form, including categories for basic information about the studies (numbing, the first author's last name and the year the study was published, and the contact information for the corresponding author), the sample sizes and grouping methods used, participant characteristics including age and gender, expressed as mean additions and subtractions above and below standard deviation and the percentages, and details of the intervention methods involved, including treatment time, the selection of acupoints, treatment efficacy, treatment cycles, side effects, and follow-up.

#### 2.3.4 Measures of treatment effect

Two authors (JR and HCF) will perform analysis independently and then cross-check treatment effect with Review Manager 5.3.5. Dichotomous data will be presented by risk ratio (RR) with 95% confidence intervals (CIs). Continuous data will be presented by mean difference (MD) or standard mean difference (SMD) with 95% CI. Other binary data will be changed into the RR form for analysis.

#### 2.3.5 Dealing with missing data

As there is possibility of missing data in literatures, we will contact the corresponding authors by email or other contacts. If the missing data are unavailable, we will analysis the existing data that is supposed as random missing.

#### 2.3.6 Assessment of heterogeneity

The heterogeneity of studies will be evaluated by *Q*-test and *I*^2^ statistic with RevMan5.3.5. The following criteria will be used: *I*^*2*^ < 50% will be deemed as low heterogeneity; *I*^*2*^ between 50% and 75% will be considered as moderate heterogeneity; *I*^*2*^ > 75% will be considered as high heterogeneity.

#### 2.3.7 Assessment of reporting bias

Publication bias and other reporting biases will be assessed by creating funnel plots. Symmetric funnel plots indicate low risk of bias, while dissymmetry ones may indicate high risk.

#### 2.3.8 Data synthesis

A meta-analysis or descriptive analysis will be performed, based on the intervention methods, the measurement methods, and heterogeneity levels, etc. If clinical and methodological heterogeneity are low, the fixed-effect model will be applied by merger analysis; the random-effects model will be applied by merger analysis when heterogeneity indicates a moderate level. If, however, a significant level of heterogeneity is found, a descriptive analysis will be performed instead.

#### 2.3.9 Subgroup analysis

Subgroup analysis will be performed based on the findings from the data synthesis, and if the heterogeneity is found to have been caused by particular features of the included studies (eg, the intervention methods [type, time, and cycle] and the measurement methods used in the clinical trials), subgroup analysis will be conducted relevant to these categories.

## 3 Discussion

Tuina is an ancient physical therapy of TCM which can be traced back to around 220 BC. With the features of simpleness, high performing and cost-effective, Tuina is widely used for myopia in ancient time and nowadays. In recent years, there have been more and more clinical reports on the treatment of myopia, but high-quality trail is still insufficient. This review will begin when necessary trails are meeting. In order to give compelling evidence and better guide in clinic practice, all actions of this review will be performed according to Cochrane Handbook 5.2.0.

## Author contributions

**Conceptualization:** Jiao Rong, Fujie Jing,Jing Li.

**Data curation:** Huichao Feng, Jing Li.

**Investigation:** Tianjiao Lu,Qian Zhuang.

**Methodology:** Jiao Rong, Xinghe Zhang.

**Supervision**: Jiao Rong, Jing Li

**Validation:** Fujie Jing, Xinghe Zhang.

**Visualization:** Jiao Rong.

**Writing–original draft:** Jiao Rong, Huichao Feng, Jing Li

**Writing–review and editing:** Fujie Jing.
